# 

**Published:** 1877-08

**Authors:** 


VOLUME XV | AUGUST—1877. | NUMBER 5.

### ATLANTA Medical and Surgical Journal.

MONTHLY: THREE DOLLARS PER ANNUM, IN ADVANCE.

EDITOR:

J. G. WESTMORELAND, M.D.

*Professor Materia Medica in Atlanta Medical College.*

ASSOCIATE EDITOR:

R. W. WESTMORELAND, M.D.,

*Assistant Demonstrator and Prosector of Anatomy in Atlanta Medical College.*

H. H. DICKSON, PUBLISHER.

*PAX ET SCIENTIA, SED VERITAS SINE TIMORE.*

ATLANTA, GEORGIA:

*H. H. DICKSON, BOOK AND JOB PRINTER AND BOOKBINDER.*

1877.

